# A large outbreak of COVID-19 in a UK prison, October 2020 to April 2021

**DOI:** 10.1017/S0950268822000991

**Published:** 2022-05-30

**Authors:** James P. Adamson, Christopher Smith, Nicole Pacchiarini, Thomas Richard Connor, Janet Wallsgrove, Ian Coles, Clare Frost, Angharad Edwards, Jaisi Sinha, Catherine Moore, Steph Perrett, Christie Craddock, Clare Sawyer, Alison Waldram, Alicia Barrasa, Daniel Rh. Thomas, Philip Daniels, Heather Lewis

**Affiliations:** 1 Centre for Disease Surveillance and Control, Public Health Wales, Cardiff, Wales, UK; 2 UK Field Epidemiology Training Programme, UK Health Security Agency, London, UK; 3 Improvement Cymru, Public Health Wales, Cardiff, Wales, UK; 4 Pathogen Genomics Unit, Public Health Wales, Cardiff, Wales, UK; 5 Cardiff University School of Biosciences, Cardiff University, Cardiff, Wales, UK; 6 Operations, ‘Prison A’, Wales, UK; 7 Microbiology, Public Health Wales, Cardiff, Wales, UK; 8 Health Protection Division, Public Health Wales, Cardiff, Wales, UK; 9 UK Health Security Agency, London, UK

**Keywords:** COVID-19, epidemiology, infectious disease epidemiology, outbreaks, public health

## Abstract

Prisons are susceptible to outbreaks. Control measures focusing on isolation and cohorting negatively affect wellbeing. We present an outbreak of coronavirus disease 2019 (COVID-19) in a large male prison in Wales, UK, October 2020 to April 2021, and discuss control measures.

We gathered case-information, including demographics, staff-residence postcode, resident cell number, work areas/dates, test results, staff interview dates/notes and resident prison-transfer dates. Epidemiological curves were mapped by prison location. Control measures included isolation (exclusion from work or cell-isolation), cohorting (new admissions and work-area groups), asymptomatic testing (case-finding), removal of communal dining and movement restrictions. Facemask use and enhanced hygiene were already in place. Whole-genome sequencing (WGS) and interviews determined the genetic relationship between cases plausibility of transmission.

Of 453 cases, 53% (*n* = 242) were staff, most aged 25–34 years (11.5% females, 27.15% males) and symptomatic (64%). Crude attack-rate was higher in staff (29%, 95% CI 26–64%) than in residents (12%, 95% CI 9–15%).

Whole-genome sequencing can help differentiate multiple introductions from person-to-person transmission in prisons. It should be introduced alongside asymptomatic testing as soon as possible to control prison outbreaks. Timely epidemiological investigation, including data visualisation, allowed dynamic risk assessment and proportionate control measures, minimising the reduction in resident welfare.

## Introduction

Prisons are crowded communal settings. During the coronavirus disease 2019 (COVID-19) pandemic, prisons have been highly susceptible to outbreaks, resulting in higher levels of morbidity and mortality in residents and staff than the general population [1–3]. During the first wave, 7.6 confirmed COVID-19 cases were reported per 1000 prison residents in England and Wales compared with 4.9 in the general population [4]. In the second wave, this rose to 75 cases per 1000 population in prisons, compared to 46 cases per 1000 overall, by the end of December 2020 [5]. Control measures focusing on isolation and cohorting were initiated in prisons in Wales [6], reducing mixing and visits. These restrictive regimes can negatively affect physical and mental wellbeing through loss of control [7–9], consequently reducing cooperation: COVID-19 caused prison unrest and rioting in Europe early in the pandemic [10]. This is concerning given prisoners have worse physical and mental health than the general population and are regularly exposed to many health risks including smoking, poor hygiene and weakened immunity [3]. Measures to limit the spread and impact of COVID-19 in Welsh prisons began several weeks before the first prison-cases were seen. On 11 February 2020, Public Health England circulated their first interim guidance for COVID-19 in prisons, which was adopted in Wales. Infection control processes were established in all Welsh prisons, continuing throughout the pandemic in line with further guidance.

In October 2020, Public Health Wales (PHW) was notified of a case of COVID-19 in a resident of a large male prison in South Wales, UK (‘Prison A’), which has approximately 1700 residents and 850 staff. This index case had a history of respiratory illness and was in the hospital at the time of notification. After a negative PCR result on hospital admission, they tested PCR positive during their stay, classifying them as a hospital-acquired case. Two weeks later, an incident management team (IMT) was convened to review 25 more cases (20 residents, 5 staff), epidemiologically linked to the index case. The IMT declared an outbreak and established an outbreak control team (OCT) which managed this outbreak through collaborative decisions, as defined in the outbreak plan for Wales [11].

## Methods

The OCT met weekly to discuss case numbers, epidemiology, control measures and operational issues related to the outbreak. The roles and organisations of OCT members are given in Table 1. We describe the epidemiology and control measures implemented for this outbreak.

**Table 1.** Overview of roles of the OCT membership, October 2020–April 2021

Organisation	Job Title	Role
Public Health Wales	Consultant in Public Health	Chair
	UK FETP Fellow	Epidemiological investigation
	Improvements Manager	Line listing management & telephone interviews
	Health Protection Nurse	Liaison with prison about cases
	Lead Nurse for Health and Justice	Providing context at an all-Wales and UK level
	Bioinformatician	WGS methods and results interpretation
Local Health Board	Consultant in Public Health	Providing local health board delivery information
Prison A	Director	Strategic & tactical intelligence about Prison A
	Deputy Director	Strategic & tactical intelligence about Prison A
	Head of Health Care	Intelligence on operational aspects of Prison A
	Clinical Lead	Intelligence on operational aspects of Prison A
	Compliance Manager	Detailed case information & operational aspects
Local Council	Environmental Health Officer	Local Government context
HMPPS Wales	Director (Strategic Support & Assurance)	Providing intelligence at a UK prisons level

UK FETP, UK Field epidemiology training programme; WGS, whole-genome sequencing; HMPPS, Her Majesty's Prisons and Probation Service.

A possible case was any staff or resident at Prison A on or after 14 October 2020 who had symptoms compatible with COVID-19 (cough and/or fever and/or loss of smell/taste), without a PCR test result. A probable case was a possible case with a positive PCR test result that had not been verified by PHW. A confirmed case was any probable case with a positive PCR test result, which had been verified by PHW. A discarded case was a possible case whose PCR test result was negative.

In accordance to then-current national guidance, symptomatic residents and staff were tested for the presence of severe acute respiratory syndrome coronavirus 2 (SARS-CoV-2) RNA using PCR tests. Asymptomatic screening was introduced in the Admissions unit (A-Block) from December 2020 (see control measures).

We telephoned all staff cases to discuss their movements and contacts prior to testing positive; no resident interviewing was done due to concerns of breaking self-isolation to access a telephone. Instead, intelligence on resident cases was obtained from prison staff. Given the restrictions of movement in place during this outbreak, this information was deemed accurate and reliable by the OCT for contact tracing.

Case data were managed using an Excel line list containing demographic information (age, sex), staff residence postcode, resident cell number, work areas/dates, laboratory results (test dates, result status, laboratory IDs, whole-genome sequencing (WGS) links), interview dates/notes and resident prison-transfer dates. Line list management was performed by a single member of staff, in frequent contact with prison, ‘Test, Trace, Protect’ (TTP – contact tracing) and laboratory colleagues.

### Epidemiological investigations

Cases were plotted on an epidemiological curve, then mapped by accommodation block and work location to show frequency and distribution over time (total and last 28 days). Prison A's accommodation is divided into 10 accommodation blocks (Box 1) and other functional areas including ‘Industries’ (where the residents of working age undertake assigned work-activities), laundry, canteen, gym and healthcare.

Box 1. Prison A accommodation description

There are approximately 1700 male residents, consisting of adult and young offenders aged 16 years and over. It has 850 staff, of whom approximately 375 are Prison Custody Officers with the balance made up of support, administration and teaching staff. Prisoners are made up of those on remand, short-term, long-term and life sentences. The residents’ accommodation is as follows:

- A Block (Admissions): where the majority of new residents stay, known as ‘receptions’. There are four units housing around 95 prisoners each
- B Block: four separate units housing around 95 prisoners each
- *C* Block: learning needs and disability unit housing approximately 75 self-contained prisoners
- D Block: (drug unit) housing around 95 self-contained prisoners
- E & G Blocks: The young persons’ unit housing around 60 15–17 year olds, separated from other units with its own gym and education facilities. It has separate staff from other units. Prisoners from this unit do not mix with adult prison units
- H Block: The safer custody unit housing around 10–20 people in a self-contained unit
- T Block: comprises six separate units housing around 400 prisoners
- *X* Block: separate from main prison with three self-contained wings housing around 350 prisoners. It has its own industries, education and gym facilities. Each unit has its own meal tables, kitchen area and association area
- The vulnerable prisoners Unit: is comprised of X3 and T6, both separate from each other and other prison units

Epidemiological curves and case location mapping were updated weekly, providing dynamic visual aids for risk assessment. Where cases were present in multiple locations, the overlap was highlighted on the maps. Test date was used for asymptomatic cases and symptom onset date used for symptomatic cases. Crude attack rates were calculated by location using prison records for residents and staff (January 2021: 1693 residents and 832 staff). Variation in attack rates was measured using a two-proportion *z* test. Finally, case hospitalisation ratios for staff and residents were calculated.

Whole-genome sequencing was carried out by the PHW Pathogens Genomics Unit (PenGU) using the ARTIC protocol for ‘Illumina’ (standard turnaround) or Oxford ‘Nanopore’ (rapid turnaround) to assess the genetic relationship between cases, aid epidemiological investigation and determine control measures. Sequencing was performed only on samples meeting set quality criteria (including a diagnostic result with a cycle threshold value of less than 30). Samples were processed using the ncov2019 ARTIC Nextflow pipeline [12] and were analysed using the CLIMB COVID analysis platform [13] making use of the civet [14] and MircoReact [15] tools for analysis and visualisation of the outbreak.

### Outbreak control measures

Certain COVID-19 control measures were in place before this outbreak, mandated across the Welsh Prison estate start of the pandemic, and vaccination was introduced for residents during it. Residents were vaccinated by age-cohort by the prison healthcare team; the first vaccination was given in January 2021. Prison staff were offered vaccination in the community by age-cohort, commensurate with the public, which Prison A's management encouraged. The most severe control-measure restrictions (‘level four-regime’, including cell-isolation) could be lifted when positivity rates were low. To confirm this, asymptomatic testing (PCR) was used on a weekly basis, piloted in staff in the vulnerable prisoners unit (VPU) from December 2020 due to the high attack-rate. Testing was extended to staff in the YPU from January 2021 and offered to all staff and residents later that month. Lateral flow device (LFD) testing began in February 2021, becoming available to all staff and residents by the end of March 2021. Details of how and where control measures were applied are described in Table 2.

**Table 2.** Control measures used at Prison A, before and during outbreak

Description	Residents	Staff	Details
Control measures already in place when outbreak declared			
Enhanced cleaning	✓	✓	Hand-washing stations and 70%-alcohol-gel dispensers installed in all areas.
Safety briefings	✓	✓	Regular reiteration of importance of hand hygiene and social distancing.
Mandatory face coverings	✓	✓	Signage installed throughout the prison; all-persons challenge to reinforce these rules.
Reduced room capacity	✓	✓	Risk assessment based on internal area of all communal/meeting rooms to allow at least 2 metres distance between people Signage stating maximum occupancy on door.
Sub-group socialisation		✓	Staggered socialisation times, when mixing was permitted, to reduce mixing and aid social distancing.
Control measures introduced during this outbreak			
Vaccination		✓	Vaccinated by age-cohort in line with general population priority (staff offered vaccination in community clinics).
Control measures introduced by the OCT			
Exclusion	✓	✓	Symptomatic staff were excluded from work, asked to take a PCR test and remained in self-isolation until the result was known. Symptomatic residents took a PCR test and remained in cell-isolation pending results. Where cells were shared, contact formed a ‘bubble’; they followed the same isolation period as cellmate, dependent on results.
Reverse cohorting		✓	New resident-admissions, from courts system or inter-prison transfer, were reverse-cohorted by date to limit transmission in either direction between people living and working in A-block.
Asymptomatic testing	✓	✓	Asymptomatic testing (day one and day five PCR tests) implemented for new resident-admissions from December 2020. Admissions grouped by admission date until 14 days after arrival before commencement of the induction-programme and relocation to another permanent residential-block. The testing team provided peripatetic testing for residents at accommodation blocks and testing for all staff based on a shift pattern.
Minimising mixing	✓	✓	To minimise new introductions and person-to-person transmission, staff worked in one area only, unless operationally necessary otherwise. Staff overtime was restricted to the same area as normal hours. Staff dining became takeaway during March 2020. Staff were frequently advised not to car share. Residents performing essential work (kitchen, cleaning and laundry) were organised into shift-groups so residents from the same accommodation unit worked together. Non-essential work was limited or stopped during level four restrictions.
Cell isolation (‘level four’ restrictions)		✓	The highest level of restrictions (‘level four’) were initiated in mid-December 2020 to control case rate the outbreak's peak This included suspension of visits, non-essential work and staff-movement across prison. Residents limited to 30-min per day to shower and exercise outside cells. Meals and purchases were brought to cells.

## Results

**Fig. 1.**
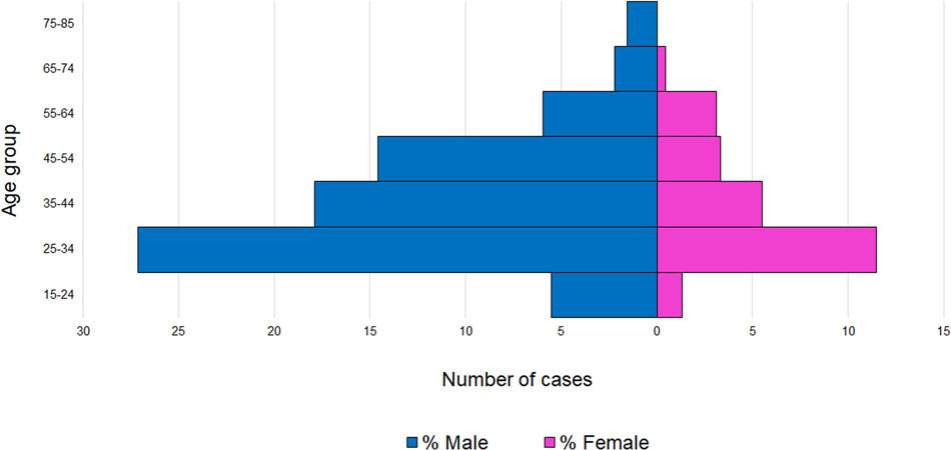
Distribution of cases by age and sex, Prison A, October 2020–April 2021 (*N* = 453).

Between October 2020 and April 2021 (189 days), Prison A reported 453 cases (staff *n* = 242, 53%; residents *n* = 211, 47%) (Table 3). Most were symptomatic (64%; staff *n* = 163, 67%; residents *n* = 126, 60%) (Table 3) with a mode of 25–34 years for both sexes (11.5% females, 27.15% males) (Fig. 1). Three staff were hospitalised due to their COVID-19 illness; case hospitalisation ratios were 0.36 for staff and 0.06 for residents. One death was linked to this outbreak; a resident with a positive PCR test result more than 28 days but fewer than 90 days before death. Telephone interviews were completed with 99% of staff cases.

**Table 3.** Overview of case types at Prison A, October 2020–April 2021 (*N* = 453)

Symptomatic asymptomatic			Total	AR%
Staff	163	79	242	29.1
Residents	126	85	211	12.5
Total	289	164	453	–

The index case was resident in A-block prior to hospital admission; 12 staff cases worked hospital bed watch shifts for the index case during their infectious period and subsequently worked in A-block, T-block, young persons' unit (YPU) and safer custody unit (SCU). However, there were already staff cases in SCU prior to these staff working back in Prison A.

Crude attack rate was higher in staff (29%, 95% CI 26–64%) than in residents (12%, 95% CI 9–15%). Accommodation-units' specific attack rates ranged from 0% to 26% in residents and 24% to 90% in staff (Table 4). Admissions (A-block), D-block, SCU and YPU experienced the highest overall attack rates (Table 4). An overview of staff cases in other work locations (Table 5) shows the highest attack rates in the testing and mentor teams.

**Table 4.** Overview of cases by accommodation units, Prison A, January 2021 (*N* = 332^†^)

Staff cases^†^					Resident cases				Total cases	
Area	Pop.	Symp.	Asymp.	AR (%)	Pop.	Symp.	Asymp.	AR (%)	N	AR (%)
A	48	22	9	64.6	355	24	65	25.1	120	29.8
B	42	10	3	31	366	9	0	2.5	22	5.4
C	17	3	1	23.5	74	0	0	0	4	4.4
D	14	8	0	57.1	91	23	1	26.4	32	30.5
SCU	10	8	1	90	12	0	1	8.3	10	45.5
T	78	15	10	32.1	410	36	4	9.8	65	13.3
X	47	10	4	29.8	325	14	11	7.7	39	10.5
YPU	44	17	21	86.4	60	0	2	3.3	40	38.5
Total	300	93	49	–	1693	106	84	–	332	–

† – Note: some staff worked in more than one location during this outbreak, so total is greater than 242.

Pop, population; Symp., symptomatic; Asymp., asymptomatic; AR, attack rate.

**Table 5.** Overview of staff cases by location or team, Prison A (*N* = 103^†^), October 2020–April 2021

Location or Team	Cases	Pop.^a^	AR^b^ %
Admin building and senior management team	8	45	17.8
Education and training teams	7	71	9.9
Gym	4	15	26.7
Healthcare	8	49	16.3
Interventions team	2	25	8.0
Mentor, families and pastoral support teams	6	30	20.0
Main industries	9	20	45.0
Main stores, facilities and waste management	20	56	35.7
Offender management unit	7	59	11.9
Security and nights teams	18	61	29.5
Testing team and pharmacy	4	11	36.4
Other^c^	10	–	–
Total	103	442	23.4

† – Note: some staff worked in more than one location during this outbreak. (Some teams have been combined to maintain anonymity).

a Pop., population of staff location of team.

b AR, attack rate.

c Other contractors or short-term staff.

**Fig. 2.**
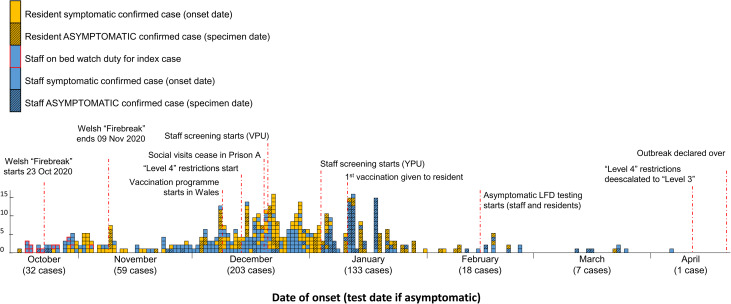
Epidemiological curve of cases, by case type, Prison A, October 2020–April 2021 (*N* = 453).

**Fig. 3.**
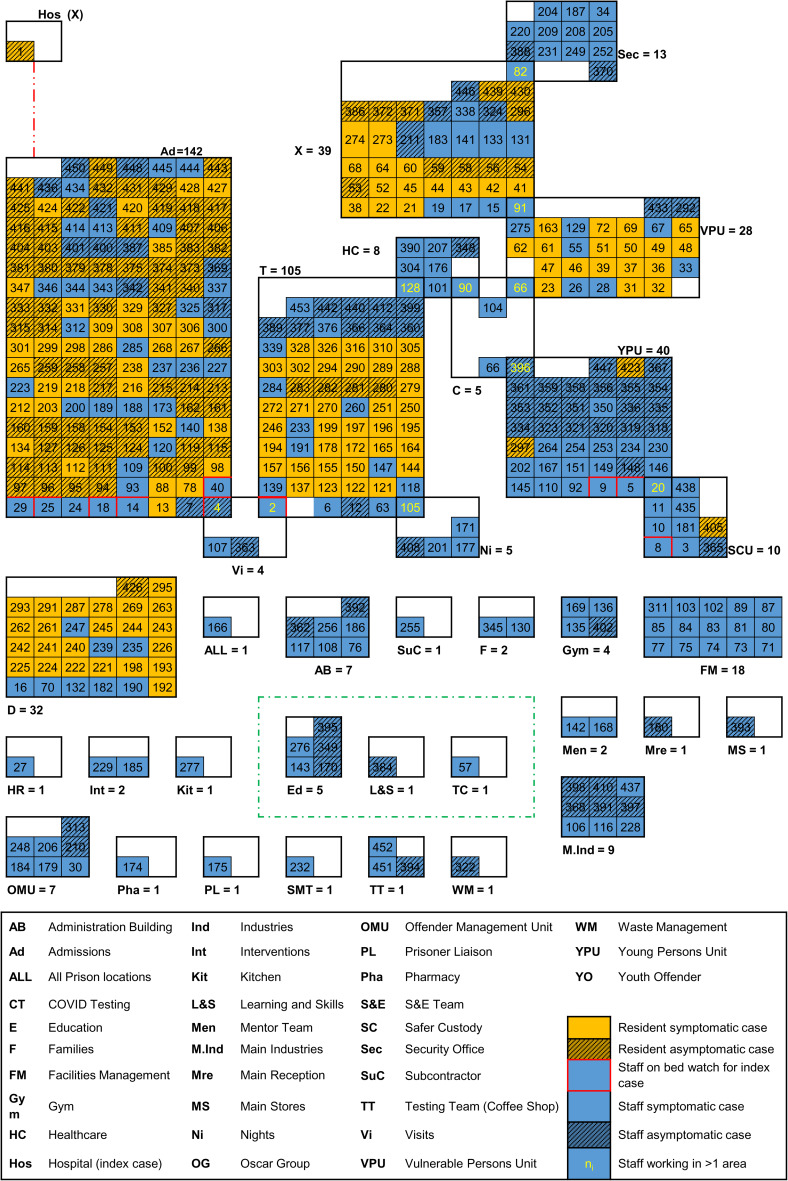
Mapping of cases, by case type and functional area, Prison A, October 2020–April 2021 (*N* = 453).

An epidemic curve (Fig. 2), highlighting dates of key control measures, shows that the first half of the outbreak (87 days) comprised 346 (76%) of the cases reported. Of these, 75 (22%) were asymptomatic. During the second half of the outbreak (86 days), 82 of the 106 cases (77%) were asymptomatic. Mapping cases by area of work or residence showed variation in attack rates by area and which cases were associated to more than one location (Fig. 3).

Sequencing of a sample from the index case and one prison staff member on bed watch for the index case revealed that the resident and staff member infections were of different lineages and not closely genetically related.

Subsequent WGS testing showed a third, different phylotype present in the prison; a cluster of seven X-block resident cases employed in the prison's print shop had identical phylotypes. One staff member's WGS result was this same phylotype; follow-up interviews revealed brief, informal contact between these cases whilst moving between locations. By the end of October 2020, 26 cases' test samples had been sequenced (15 residents, 11 staff), identifying 11 different phylotypes. Phylotypes are based upon the position of a sample on the global SARS-CoV-2 phylogenetic tree, and where two cases possess phylotypes that differ, it is possible to exclude those two cases from being a directly linked transmission. Where two cases possess the same phylotype, when combined with other epidemiological data, it is possible to conclude the cases are part of a transmission group. Taking these WGS results with the other epidemiological data, WGS demonstrated that there had been multiple introductions into the prison as well as subsequent person-to-person transmission.

**Fig. 4.**
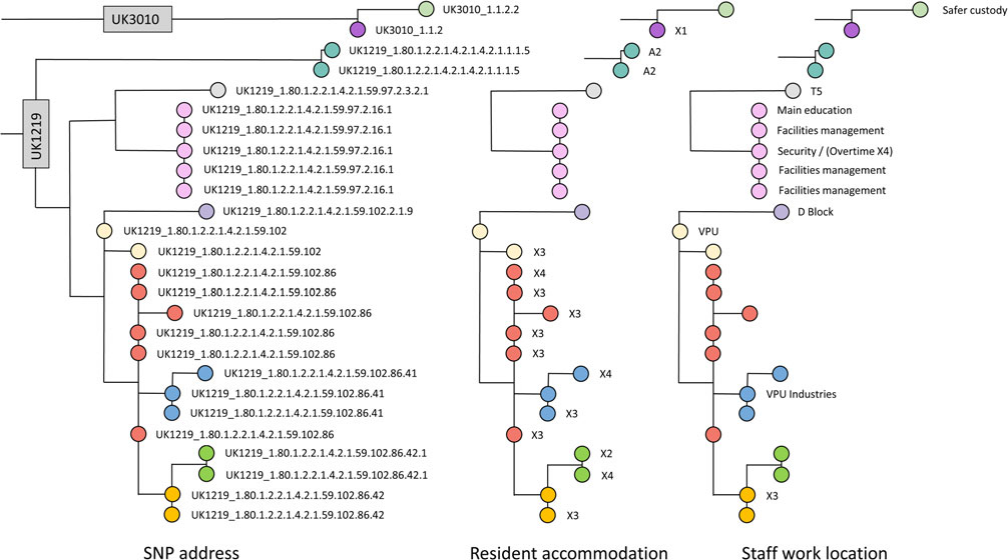
Distribution of staff and resident cases by phylotype address, resident accommodation and staff work location, Prison A, November 2020 (*N* = 26).

WGS enabled a delineation of person-to-person spread, and helped demonstrate that this had occurred between residents and staff through identical phylotype results by time, place and person. Person-to-person spread was also found in the same way between staff working in the same location with no contact to residents (Fig. 4). Clusters by team, such as in Estates and Facilities Management, were examples of where keeping two metres apart was often difficult when completing essential duties.

## Discussion

We describe a large, long-running outbreak of COVID-19 affecting 211 prison residents and 242 staff. During the second peak in Wales in December 2020, there were 102 resident cases and 101 staff cases in this outbreak, representing a period-incidence of 60.4 cases per 1000 population (residents) and 118.8 cases per 1000 population (staff) respectively. This was higher for staff but lower for prisoners compared to the England and Wales prison average in the same period, highlighting the impact of community incidence on introductions to the prison. The proportion of cases hospitalised was six-times higher in staff than residents, highlighting potential differences in self-perception of disease severity or inequity of access to healthcare.

There were notable differences in infection patterns; YPU cases were mostly staff (38/40; 95%) and both resident infections were asymptomatic, consistent to community infections whereby young people generally experienced milder or no symptoms of COVID-19. Conversely, D-block cases were mostly residents (24/32; 75%) which may highlight difficulties in social-distancing for those with disabilities. The accommodation with the highest attack rates (SCU = 45.5%; YPU = 38.5%; D = 30.5%) were all self-contained units with fewer residents and staff; thus each new case contributed a higher weighting to attack rates than the larger accommodation units.

Where staff had greater exposure to others, attack rates were higher; exemplified by the Mentor and Testing Teams experiencing attack rates of 50%. These roles involved contact with many people and, for the Testing Team, an obvious and repeated risk of contact with infectious asymptomatic cases.

Because some staff-teams were small and others worked in more than one area, attack rates should be interpreted with caution. What is clear is that the 242 staff cases in a stable population of 850 *vs.* 211 resident cases in a fluctuating population of over 1690 during this outbreak, means the attack rate was far higher in staff than residents (Overall AR = 29% in staff *vs.* 12% in residents). Attack rates could have been affected by testing policy; staff were able to access free testing in the community as frequently as they chose for the entirety of the outbreak (PCR and then LFD) whereas residents had to report symptoms and request a test. Residents might also have been reluctant to report symptoms knowing this would incur cell-isolation. The skew in male cases reflects the resident population and the majority of staff being male. The higher proportion of staff cases in this outbreak reached statistical significance. One explanation for this could be the long periods of resident cell-isolation and greatly reduced contact opportunities during socialisation times. Furthermore, staff mixed with others outside work, which might not have been detected by this investigation, despite interviewing. Contacts of cases in Wales were required to self-isolate from 1 June 2020. Testing was voluntary and results could be notified by phone or on the NHS COVID-19 app to aid contact tracing. Obviously, residents in Prison A could not use mobile phones so contact tracing relied on intelligence from staff and residents regarding people's movements. Testing of contacts of cases in the community was introduced in Wales on 10 March 2021, towards the end of this outbreak, by which time asymptomatic testing had already been rolled out in Prison A in line with other institutional settings.

Effective outbreak management requires preventative and reactive measures and a collaborative, multi-agency OCT that acts at all policy and operational levels, gathering accurate information and implementing appropriate and timely control measures. The principal control measures instigated by the OCT can be categorised as standard-unilateral measures and targeted-proportional measures. The latter expedited relaxation of other controls imposed based on evidence of reduced infection and transmission.

**Fig. 5.**
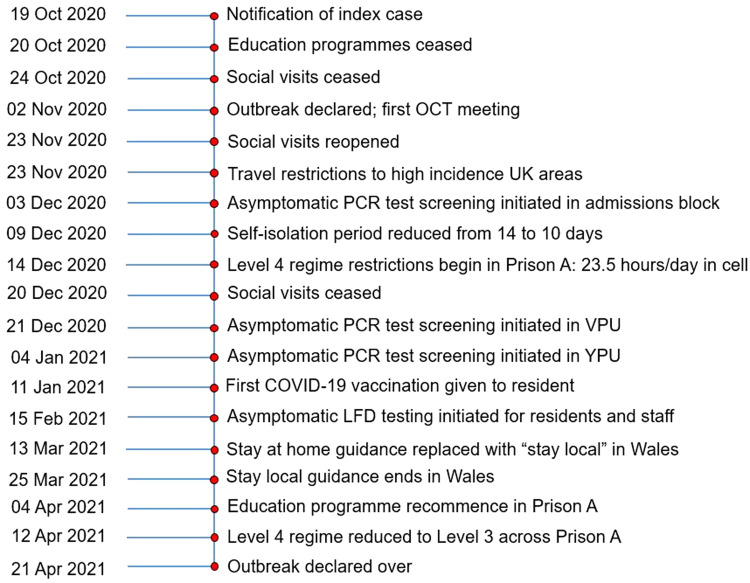
Timeline of Prison A outbreak, October 2020–April 2021, from notification of first case to declaration of end of outbreak.

Cohorting and testing new-admissions reduced their ability to infect others. Asymptomatic screening identified cases who would otherwise have remained an infection risk and highlighted additional efforts required to end level four regime measures safely in specific areas. The timeline of the outbreak and key dates and control measures is shown in detail in Figure 5.

This OCT benefitted from dedicated and consistent epidemiological support, line listing management and case distribution mapping. The importance placed on an epidemiological investigation by the OCT chair improved its ability to determine and expedite control measures through a combination of case-location mapping and WGS results. Mapping revealed where further investigation and control measures were required and allowed phased relaxation of restrictions such as cell-isolation. WGS turnaround was two to seven (median = three) days and showed this outbreak was seeded by multiple introductions to the prison but also involved person-to-person transmission. In terms of the initial X-block cluster identified by WGS, the same phylotype was widely distributed in communities in Wales at this time in the pandemic and many staff were resident in these areas, giving rise to a plausible introduction-pathway.

Early or temporary release of prisoners was discussed widely early in the pandemic [16], although little evidence suggests this happened at scale anywhere. Cohorting and reduced mixing worked well in this outbreak. However, residents undertake essential services (cooking, laundry, cleaning) resulting in mixing. Prisons have a continuous churn of residents; despite limiting transfers during the peak incidence of COVID-19 in the UK, they were not stopped, resulting in residual risk of infection. Mass screening is effective in identifying cases and limiting the spread of disease [17] and was employed here to minimise transmission between groups and identify cases who would have otherwise have remained an infection risk. Scaling-up testing capacity to all staff and residents demonstrated the majority of cases were asymptomatic in the second-half of this outbreak (106 cases in 86 days, 18 symptomatic). If other asymptomatic cases had been identified in this way at the start of this outbreak, its duration might have been reduced. It also highlights inequalities in testing practice and suggests the epidemic curve is not a true reflection of case rates by location, especially in the first-half of this outbreak. The collaborative OCT approach allowed them to discuss, consider, adapt and react within the changing guidance-landscape of the COVID-19 pandemic [18].

Prisons, by design, control activities and movement but provide little personal space. Compared to the wider community, this complicated the control of COVID-19 spreading in a naïve population. As such, stringent social distancing measures were deemed necessary to control COVID-19 in Prison A. These measures were effective and likely reduced transmission to adjacent shared areas and within cultural groups, as reported in other prison outbreaks [19].

Integration of epidemiological techniques with WGS allowed the OCT to make balanced risk assessments with clarity about the direction and intensity of transmission events. As case-rates were reduced in a particular location, relaxation of control measures followed. The social distancing and enhanced hygiene campaigns in place for eight months before this outbreak clarified the impact of the OCT's control measures.

Vaccination is a key control measure strategy for many infectious diseases. There was much debate about vaccination prioritisation for prison residents and staff given the high propensity for transmission. This did not happen in prisons in England and Wales. Modelling has shown that vaccinating everyone living and working in prisons is the most effective strategy for reducing COVID-19 cases, transmission and outbreaks, with an 89% reduction in cases over three years [2] and would yield a similar impact to restrictive and intensive control measures [20]. The Joint Committee on Vaccination and Immunisation (JCVI) considered this twice but it did not support prioritising this population [20, 21]. The combination of high vaccine coverage with other non-pharmaceutical interventions was shown to reduce cumulative infections by up to 54% in another model [22]. Uptake figures for resident vaccination is affected by prison churn (amongst other things); COVID-19 vaccinations had been given to the majority of those requesting it in Prison A post outbreak (July and November 2021, Table 6).

**Table 6.** Resident COVID-19 vaccination status overview, Prison A (July and November 2021)

	July 2021	November 2021
Total resident population	1639	1591
Vaccines given	1555	2300
One vaccine dose	1052	1122
Two vaccine doses	518	1090
Refusals	368	464
Waiting to have first vaccine dose	219	21

Our methods had several limitations. Different testing pathways for staff and residents limited the number of samples available for WGS, particularly early in this outbreak. Additional, early WGS intelligence might have identified other transmission routes, especially in terms of community introduction via staff and inter-prison introduction via A-block. However, there was huge pressure on WGS services due to the COVID-19 pandemic, and it wasn't until January 2021 that issues around accessing and sequencing Welsh Pillar 2 samples tested by Lighthouse Laboratories were largely resolved. We only considered activities and movements of staff and residents inside Prison A; it is likely that staff socialised outside Prison A, potentially in contact with asymptomatic cases. Telephone interviews with staff cases investigated plausible vehicles of transmission, such as household contacts, but asymptomatic spread in their communities was not investigated and would have been difficult to measure. Despite the agreement of the OCT members, not interviewing resident cases was an information bias and could have revealed additional information relevant to the investigation and control measures. Lack of ascertainment of resident denominators by accommodation area due to prison churn meant comparing staff and resident attack rates was not regularly possible.

We conclude that sufficient epidemiological capacity to thoroughly investigate outbreaks allows OCTs to assess changes and implement appropriate control measures. Traditional epidemiological investigations can be augmented with WGS to determine the phylogeny of infections and inform the epidemiological plausibility of community *vs.* prison transmission. Analysis of epidemiological investigation findings revealed that admissions-block was a persistent source of infections; particular attention should be given to admissions screening. Case-distribution location mapping tracked infection progression, visualising data and allowing easy interpretation to make timely control measure responses. Mapping was equally informative for deciding when to relax control measures safely to improve resident welfare.

Limiting staff movements to one area negatively affected prison operational capacity as more staff became absent due to infection, but helped reduce further transmission. Frequent communication of COVID-19 infection control measures to all staff and residents built an inclusive culture of behaviour applicable to all staff and residents.

COVID-19 is likely to be in circulation for several years. Given the vulnerability of prison residents to infectious diseases, the prioritisation of prisoners in COVID-19 vaccination campaigns should be considered.

## Recommendations

1. Sufficient capacity for thorough epidemiological investigations is important for providing timely information for the OCT to determine actions
2. Data visualisation through case-distribution location mapping improves the speed of interpretation, aiding risk assessment to determine control measures
3. WGS is a powerful tool in assessing the plausibility of transmission chains and should be introduced as soon as possible in prison outbreak investigations where quick turnaround times are feasible
4. Cohorting, particularly in admissions blocks, is effective in limiting the transmission of COVID-19 in prisons
5. Asymptomatic testing is highly effective in identifying cases and can limit transmission events by having them isolate

## Data Availability

The data used in this investigation contain personal identifiable information of a vulnerable population. Anonymised information, including that contained in the supplementary information, required to reproduce these results is available from the corresponding author on reasonable request.

## Supplementary material

For supplementary material accompanying this paper visit https://doi.org/10.1017/S0950268822000991. click here to view supplementary material
