# Supplementary material for: A large outbreak of COVID-19 in a UK prison, October 2020 to April 2021
Source: Epidemiol Infect. 2022 May 30;150:e134. doi: 10.1017/S0950268822000991 (PMC9304949; doi:10.1017/S0950268822000991)
Supplement: Supplementary file 1 [file hygsup.zip › S0950268822000991sup002.docx]

**A large outbreak of COVID-19 in a UK prison, October 2020 to April 2021**

**ORCID IDs**

**Author information**

| **Name** | **Job title** | **ORCID** |
| --- | --- | --- |
| James P. Adamson | Field Epidemiology Training Programme Fellow | 0000-0001-9175-8287 |
| Christopher Smith | Improvement Manager, Public Health Wales, Cardiff, UK  [Christopher.Smith4@wales.nhs.uk](mailto:Christopher.Smith4@wales.nhs.uk) | 0000-0003-1544-1290 |
| Nicole Pacchiarini | Bioinformatician, Public Health Wales, Cardiff, UK  [Nicole.Pacchiarini@wales.nhs.uk](mailto:Nicole.Pacchiarini@wales.nhs.uk) | 0000-0001-5383-6053 |
| Thomas Richard Connor | Bioinformatics Lead for Pathogen Genomics, Public Health Wales, Cardiff, UK and Professor of Pathogen Genomics and Bioinformatics, Cardiff University School of Biosciences, Cardiff University, UK.  [Tom.Connor1@wales.nhs.uk](mailto:Tom.Connor1@wales.nhs.uk) | 0000-0003-2394-6504 |
| Janet Wallsgrove | Director, “Prison A”, UK  [janet.wallsgrove@uk.g4s.com](mailto:janet.wallsgrove@uk.g4s.com) | 0000-0002-5183-5978 |
| Ian Coles | Deputy Director, “Prison A”, UK  [ian.coles@uk.g4s.com](mailto:ian.coles@uk.g4s.com) | 0000-0001-8921-8687 |
| Clare Frost | Head of Healthcare, “Prison A”, UK  [clare.frost@uk.g4s.com](mailto:clare.frost@uk.g4s.com) | 0000-0002-2768-2860 |
| Angharad Edwards | Compliance Manager, “Prison A”, UK  [angharad.edwards@uk.g4s.com](mailto:angharad.edwards@uk.g4s.com) | 0000-0002-3141-5343 |
| Catherine Moore | Consultant Clinical Scientist, Public Health Wales, Cardiff, UK  [Catherine.Moore2@wales.nhs.uk](mailto:Catherine.Moore2@wales.nhs.uk) | 0000-0001-8934-0930 |
| Jaisi Sinha | Consultant in Microbiology and Virology, Public Health Wales, Cardiff, UK  [Jaisi.Sinha@wales.nhs.uk](mailto:Jaisi.Sinha@wales.nhs.uk) | 0000-0003-1056-7185 |
| Steph Perrett | Lead Nurse for Health and Justice, Public Health Wales, Cardiff, UK  [Stephanie.Perrett@wales.nhs.uk](mailto:Stephanie.Perrett@wales.nhs.uk) | 0000-0003-4372-7384 |
| Christie Craddock | Senior Nurse for Health and Justice, Public Health Wales, Cardiff, UK  [Christie.Craddock@wales.nhs.uk](mailto:Christie.Craddock@wales.nhs.uk) |  |
| Clare Sawyer | FETP Fellow, Public Health Wales, Cardiff, UK  [Clare.Sawyer2@wales.nhs.uk](mailto:Clare.Sawyer2@wales.nhs.uk) | 0000-0003-2679-7217 |
| Alison Waldram | Senior Epidemiologist and UK FETP Scientific Coordinator, London, UK  [Alison.Waldram@phe.gov.uk](mailto:Alison.Waldram@phe.gov.uk) | 0000-0002-2555-0661 |
| Alicia Barrasa | Senior Epidemiologist and UK FETP Scientific Coordinator, London, UK  [Alicia.BarrasaBlanco@phe.gov.uk](mailto:Alicia.BarrasaBlanco@phe.gov.uk) | 0000-0002-7764-554X |
| Daniel Thomas | Consultant in Epidemiologist, Public Health Wales, Cardiff, UK  [Daniel.Thomas@wales.nhs.uk](mailto:Daniel.Thomas@wales.nhs.uk) | 0000-0002-2426-5893 |
| Philip Daniels | Consultant in Public Health, Public Health Wales, Cardiff, UK  [Philip.Daniels2@wales.nhs.uk](mailto:Philip.Daniels2@wales.nhs.uk) | 0000-0002-6260-3523 |
| Heather Lewis | Consultant in Public Health, Public Health Wales, Cardiff, UK  [Heather.Lewis4@wales.nhs.uk](mailto:Heather.Lewis4@wales.nhs.uk) | 0000-0001-9660-7103 |
|  |  |  |
